# Draft Genome Sequences of *Burkholderia pseudomallei* and *Staphylococcus aureus*, Isolated from a Patient with Chronic Rhinosinusitis

**DOI:** 10.1128/genomeA.01075-15

**Published:** 2015-10-01

**Authors:** Hanna E. Sidjabat, Kyra Cottrell, Anders Cervin

**Affiliations:** a University of Queensland, UQ Centre for Clinical Research, Brisbane, Australia; b University of Queensland, School of Medicine, Brisbane, Australia; c Department of Otolaryngology, Head and Neck Surgery, Royal Brisbane and Women’s Hospital, Brisbane, Australia

## Abstract

Here, we report the draft genome sequences of *Burkholderia pseudomallei* and *Staphylococcus aureus* causing chronic rhinosinusitis. Whole-genome sequencing determined the *B. pseudomallei* as sequence type (ST) 1381 and the *S. aureus* as ST8. *B. pseudomallei* possessed the *bla*_OXA-59_ gene. This study illustrates the potential emergence of *B. pseudomallei* in cases of chronic rhinosinusitis.

## GENOME ANNOUNCEMENT

*Burkholderia pseudomallei* is a Gram-negative soil- and water-associated pathogen causing melioidosis (1). In our region, *B. pseudomallei* is more commonly found in tropical parts of Australia (2). *Burkholderia* spp. were occasionally isolated from cystic fibrosis patients (3, 4). Recently, isolation of the *Burkholderia cepacia* complex was reported among patients with nonpolypoid chronic rhinosinusitis (5). Here, we report the draft genome sequences of *B. pseudomallei* and *Staphylococcus aureus*, isolated from a patient with chronic rhinosinusitis.

A 59-year-old woman with a history of arthritis suffered from chronic rhinosinusitis with purulent discharge and retro-orbital pain over the past 3 years. The patient was regularly in contact with soil due to her gardening activities. The patient underwent sinus surgery for 3 consecutive years, from 2012 to 2014. Heavy and nearly pure growth of *Burkholderia* spp. (HS-CRS-17A) type of colonies grew from the nasal swab obtained in March 2015. Light growth of *S. aureus* (HS-CRS-17B) was identified from the same specimen.

Both *Burkholderia* spp. and *S. aureus* were subjected to whole-genome sequencing. The DNA was extracted using Ultraclean DNA extraction kit (Mobio, USA). Nextera XT DNA sample preparation kit (Illumina, USA) was used to prepare the library and the HiSeq 2000 platform for whole-genome sequencing using a previously described method (6). The CLC genomic workbench version 8.0 (CLC Bio, Aarhus, Denmark) was used for *de novo* assembly using minimum 600-bp thresholds, resulting in 269 and 60 contigs in *Burkholderia* spp. and *S. aureus*, respectively. The draft genomes of the two isolates consisted of 7,240,926 bp and 2,734,587 nucleotides, respectively. The draft genomes were annotated using Rapid Annotations using Subsystems Technology (RAST) (7). RAST annotation identified that *Burkholderia mallei* ATCC 23344 (score 530) and *Burkholderia pseudomallei* 1026a (score 504) were the two closest neighbors of our *B. pseudomallei*. *S. aureus* NN50 (score 519) and *S. aureus* T0131 were the two closest neighbors of our *S. aureus*.

The *in silico* species identification using *rspU* of HS-CRS-17A was 100% identical to *B. pseudomallei*. *In silico* MLST analysis (http://bpseudomallei.mlst.net) determined a novel ST, *B. pseudomallei* ST1381. The alleles were as follows: *ace* (8), *gltb* (1), *gmhd* (3), *lepa* (2), *lipa* (1), *nark* (2), and *ndh* (1). An oxacillinase gene, *bla*_OXA-59_, was identified in *B. pseudomallei*. *In silico* analysis of *S. aureus* MLST (http://saureus.mlst.net) identified *S. aureus* HS-CRS-17B as sequence type (ST) 8. The alleles were as follows, *arcc* (3), *aroe* (3), *glpf* (1), *gmk* (1), *pta* (4), *tpi* (4), and *yqil* (3). Of note, *S. aureus* ST8 has been reported previously among the indigenous population in Australia (8). The Panton-Valentine leucocidin (PVL) gene, which often causes serious soft tissue infection, was not detected in *S. aureus* HS-CRS-17B.

There have been approximately 200 published *Burkholderia pseudomallei* genomes (http://www.ncbi.nlm.nih.gov/genome/?term=burkholderia+pseudomallei), including genomes of *B. pseudomallei* from Australia (9–12). Most of the draft genomes were isolates from environmental and cystic fibrosis patients (9–12). Our study illustrates the emergence of *Burkholderia* spp. among chronic rhinosinusitis patients in Australia. The prevalence of *Burkholderia* spp. among patients with chronic rhinosinusitis and the need for accurate species identification warrant further investigation in our region.

### Nucleotide sequence accession numbers.

This project is registered as BioProject PRJNA291304. The BioSample numbers of *B. pseudomallei* HS-CRS-17A ST1381 and *S. aureus* HS-CRS-17B ST8 are SAMN03938520 and SAMN03938521, respectively. The GenBank accession numbers are LHQO00000000 and LGVN00000000 for *B. pseudomallei* HS-CRS-17A ST1381 and *S. aureus* HS-CRS-17B ST8, respectively.
